# Study protocol of a randomized controlled trial to test the effect of a smartphone application on oral-health behavior and oral hygiene in adolescents with fixed orthodontic appliances

**DOI:** 10.1186/s12903-018-0475-9

**Published:** 2018-02-07

**Authors:** Janneke F. M. Scheerman, Berno van Meijel, Pepijn van Empelen, Gem J. C. Kramer, Gijsbert H. W. Verrips, Amir H. Pakpour, Matheus C. T. Van den Braak, Cor van Loveren

**Affiliations:** 1 Department of Preventive Dentistry, Academic Centre for Dentistry Amsterdam (ACTA), Amsterdam, the Netherlands; 2 Department of Oral Hygiene, Inholland University, Amsterdam, the Netherlands; 3 Department of Child Health, Netherlands Organization for Applied Scientific Research (TNO), Leiden, the Netherlands; 4 Department of Health, Sports & Welfare/Cluster Nursing, Inholland University of Applied Sciences, Amsterdam, the Netherlands; 5 Department of Psychiatry and EMGO Institute for Health and Care Research, VU University Medical Centre, Amsterdam, the Netherlands; 6 Parnassia Psychiatric Institute, Parnassia Academy, The Hague, the Netherlands; 7 Department of Orthodontics, Academic Centre of Dentistry Amsterdam, Amsterdam, the Netherlands; 8 Department of Social Determinants of Health, Research Centre (SHD), Qazvin University of Medical Sciences, Qazvin, Iran; 9 Department of Nursing, School of Health and Welfare, Jönköping University, Jönköping, Sweden; 10 Department of Natural Science and Biomedicine, Centre of Oral Health, School of Health Sciences, Jönköping University, Jönköping, Sweden; 11 Department of Preventive Dentistry, Academic Centre for Dentistry Amsterdam, ACTA University, Gustav Mahlerlaan 3004, 1081 LA Amsterdam, The Netherlands

**Keywords:** Study protocol, Behavioral intervention, App, M-health, Prevention, Oral health promotion, Oral health behavior, Oral hygiene

## Abstract

**Background:**

Adolescents with fixed orthodontic appliances are at high risk of developing dental caries. To date, new smartphone technologies have seldom been used to support them in the preventive behavior that can help prevent dental caries. After an intervention-mapping process, we developed a smartphone application (the WhiteTeeth app) for preventing dental caries through improved oral-health behavior and oral hygiene. The app, which is intended to be used at home, will help adolescents with fixed orthodontic appliances perform their oral self-care behavior. The app is based on the Health Action Process Approach (HAPA) theory, and incorporates several behavior-change techniques that target the psychosocial factors of oral-health behavior. This article describes the protocol of a randomized controlled trial (RCT) to evaluate the effects of the WhiteTeeth app on oral-health behavior and oral-hygiene outcomes (presence of dental plaque and gingival bleeding) compared with those of care as usual, in patients aged 12–16 with fixed orthodontic appliances.

**Methods/design:**

The RCT has two conditions: an experimental group that will receive the WhiteTeeth app in addition to care as usual, and a control group that will only receive care as usual. Care as usual will include routine oral-health education and instruction at orthodontic check-ups. In the western part of the Netherlands 146 participants will be recruited from four orthodontic clinics. Data will be collected during three orthodontic check-ups: baseline (T0), 6 weeks of follow-up (T1) and 12 weeks of follow-up (T2). The primary study outcomes are the presence of dental plaque (measured with a modified Silness and Loë Plaque Index); and gingival bleeding (measured with the Bleeding on Marginal Probing Index). Secondary outcomes include changes in self-reported oral-health behaviors and its psychosocial factors identified by the HAPA theory, such as outcome expectancies, intention, action self-efficacy, coping planning and action control.

**Discussion:**

Since the intervention was designed to target psychosocial factors in the motivational and volitional components of the behavior-change process, we hypothesize that the app will cause greater improvements in oral-health behavior and oral hygiene more than traditional oral-health-promotion programs (i.e., care as usual).

**Trial registration.:**

The trial has been registered with the Dutch Trial Register (NTR6206: 20 February 2017).

**Electronic supplementary material:**

The online version of this article (10.1186/s12903-018-0475-9) contains supplementary material, which is available to authorized users.

## Background

In 2011, 60% of young Dutch adults had had orthodontic treatment during adolescence [1]. Despite its functional and esthetic benefits, fixed-appliances therapy is associated with an increased risk of dental caries, mainly in the form of white-spot lesions, whose estimated prevalence ranges from 50 to 90% [2–5]. After orthodontic treatment, the lesions can become an esthetic problem, and may progress to more extended caries lesions [6, 7].

Although fluoride administration (e.g. fluoride mouth rinses) and the regular and effective removal of plaque from all tooth surfaces are essential to preventing oral diseases [8, 9], patients undergoing fixed orthodontic treatment have difficulty reducing dental plaque, as fixed orthodontic appliances (brackets) impede cleaning [10]. Previous studies also showed that only 50% of patients used fluoride mouth rinses as prescribed [11, 12]. Over 60% of orthodontists in the Netherlands stated that patients’ oral hygiene deteriorates during orthodontic treatment [13]. A majority of orthodontists indicated that 5–10% of patients interrupt orthodontic treatment prematurely due to poor oral hygiene [13, 14]. A recent cross-sectional study in the Dutch city of Almere showed that most young orthodontic patients had low levels of oral hygiene and did not follow oral-health recommendations [15]. This emphasizes the need for intervention strategies to improve oral hygiene in young people with such appliances.

Today’s use of smartphones offers new opportunities for developing oral-health interventions. This high use – particularly by young people – could ensure comprehensive access to adolescents with fixed orthodontic appliances [16–18]. Smartphones may thus be an appropriate medium for providing oral-health care information, changing oral-health behavior and improving oral hygiene.

For this reason, we used the intervention-mapping protocol [19] to develop a smartphone application (the WhiteTeeth app) intended to prevent dental caries by improving adolescents’ oral-health behavior and oral hygiene during fixed orthodontic treatment. As part of the intervention mapping process, we conducted a systematic review with meta-analysis and a cross-sectional study in which we analyzed oral-health behaviors and psychosocial factors (i.e. intervention targets) in adolescents [15, 20]. The results of this suggested that the Health Action Process Approach (HAPA) theory would be an appropriate theory for underpinning the present intervention [21]. Incorporating several behavior-change techniques that target the psychosocial factors identified by the HAPA theory, the WhiteTeeth app thus focuses (1) on controlling dental plaque levels through improved dental cleaning and (2) on increasing the use of fluoride mouth rinse.

In this article we describe the design of a randomized controlled trial (RCT) to compare the effectiveness of the WhiteTeeth app with that of care as usual. The primary objective of the study is to determine whether the use of the WhiteTeeth app by orthodontic patients aged 12 to 16 improves oral-health behavior and oral hygiene. The primary outcomes of this RCT are changes in dental plaque levels, and gingival bleeding upon marginal probing. The secondary outcomes are changes in oral-health behaviors, including tooth-brushing and the use of dental-cleaning aids and fluoride mouth rinse, and psychosocial factors of oral-health behavior. We will test the mediating effects of psychosocial factors on changes in oral-health behaviors and oral hygiene. Our hypothesis is that the use of the WhiteTeeth app in the intervention group will improve oral-health behavior and oral hygiene more than usual care does (the control group). We expect changes in the psychosocial factors to be associated with the factual changes in oral-health behavior.

## Methods/design

**Fig. 1:**
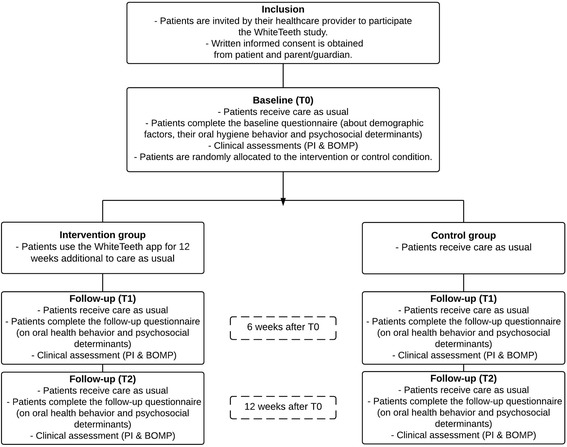
An overview of the study procedures and design

This study is a multicenter, parallel, randomized controlled trial with two conditions: an experimental group that will receive the WhiteTeeth app in addition to care as usual, and a control group that will receive only care as usual. Data will be collected during three orthodontic check-ups: baseline (T0), 6 weeks of follow up (T1) and 12 weeks of follow up (T2) (Fig. 1).

### Participants and recruitment

One hundred forty-six orthodontic patients aged 12 to 16 will be recruited in four orthodontic clinics in the western Netherlands. The study sites will be eligible if (1) standard oral-health instructions are administered according to the clinical guidelines of the department of orthodontics at the Academic Centre for Dentistry Amsterdam (ACTA) (See paragraph ‘Care as usual’); if (2) the clinicians are orthodontists registered in the Dutch orthodontic specialist register, or are postgraduate orthodontic students supervised by a registered orthodontist; if (3) the orthodontists are willing not to change their method of providing oral-health education or instructions during the study period (care as usual); if (4) there is scope for the researcher to inform patients about treatment allocation and the app (in the case of participation in the intervention group); and if a dental chair is available for the oral-health assessments. We will include the same number of participants from each study site.

Before the study starts, a presentation on it will be given at the eligible clinics. Patients who meet the eligibility criteria (Table 1) will be informed about the study by their clinician. They will then receive an information letter and an informed consent form (see Additional file 1), and have two visits before the baseline measurements. The letter will include information on the intervention, the study design, and ethics. Patients will be recruited over a 3-month period. The baseline assessment will be scheduled after patients’ and their parents’ written informed consent has been received.

**Table 1:** Eligibility criteria

Patients will be eligible for the study if they meet the following criteria:
- Boys or girls aged 12 to 16.
- For at least 6 weeks, they must have maxillary and mandibular fixed orthodontic appliance therapy, which consists of bonding at least premolar-to-premolar with edgewise appliances and their modifications.
- They have not been scheduled for removal of fixed orthodontic treatment before the end of the study.
- They have no physical and/or mental disabilities that will impede their ability to perform their own oral hygiene activities.
- They are not engaged in other oral-health education or research program.
- They do not have enamel and dentine dysplasia and/or craniofacial malformation (e.g. cleft).
- They have a sufficient command of the Dutch language.
- They possess a smartphone with software IOS ≥7 or Android ≥4.1.
- Patients and their parents are able or willing to give informed consent.
- Patients must not use medication that may affect plaque accumulation, for example antibiotics and antibacterial mouth rinses.

### Randomization

Randomization to the intervention group or control group will be performed at the patient level. A co-author who is not involved in data collection or analysis will use a random sequence generator (http://www.random.org) to allocate patients in a random sequence to the intervention or control group. In a separate room after completion of the baseline measurements, an independent researcher will tell individual participants to which group they have been allocated. If this is the intervention group, the researcher will help them install and unlock the app on their smartphone, and will also provide information on how to use it. To identify dental plaque during the intervention period, participants in the intervention group will receive twelve disclosing tablets (Gum® Red-Cote®). To prevent treatment contamination, adolescents in the control group will not have access to the intervention, as the app will be locked with a personal code that will only be provided to the adolescents in the experimental group.

### Care as usual

Care as usual will be provided according to the orthodontic protocol at the Academic Centre for Dentistry Amsterdam (ACTA). Whether in the intervention group or control group, all patients will receive oral dental-health education and instructions on plaque control at each visit.

Approximately every 6 weeks, orthodontists will invite their patients for a check-up visit, when they will adjust the braces, evaluate dental hygiene and the progress of the teeth, and, if necessary, provide oral-health education. On the basis of the ACTA guidelines, they will recommend the following oral-health behavior to their patients.

First, to control dental plaque levels, patients will be recommended to brush their teeth with fluoride-containing toothpaste at least twice a day according to the 5-step method. Thus, (1) after brushing the gingival sites, they should brush (2) above and then (3) under the bracket on the buccal sites of the teeth. They should end by brushing (4) the occlusal sites and (5) the lingual or palatinal sites of the teeth. These five steps take approximately three minutes to fulfill [11].

Next, to ensure proper plaque removal, the orthodontists will recommend the use of dental aids (e.g. a proxy brush to clean the tooth surfaces around the brackets). To prevent dental caries during orthodontic treatment, they will also highly recommend the daily use of fluoride-containing mouth rinse and toothpaste [9, 11]. Finally, they will recommend patients to limit their consumption of sugars, refined carbohydrates and acid drinks/soft drinks [22].

### The behavioral intervention: WhiteTeeth app

**Fig. 2:**
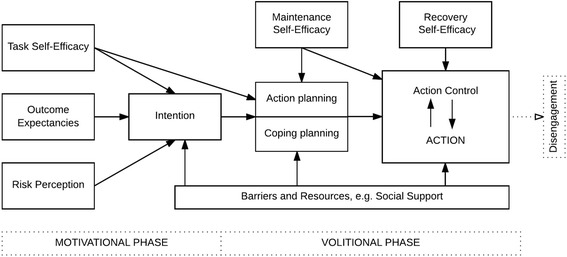
The Health Action Process Approach Model [21]

The app is intended for use at home as an add-on intervention to care as usual. The intervention is based on the Health Action Process Approach (HAPA) theory (Fig. 2), which has demonstrated its usefulness to understanding oral hygiene behaviors in adolescents with fixed orthodontic appliances [15, 21]. The theory classifies the establishment of behavior into two phases: a motivational, intention-forming phase, and a volitional phase in which intention is translated into action [21]. The WhiteTeeth app integrates several behavior-change techniques (BCTs) that target these motivational and volitional behavior-change processes [23, 24]. BCTs addressing the factors of the motivational phase include: providing information on health consequences, visualizing dental plaque with disclosing tablets [25], and demonstrating the desired behavior [24]. BCTs addressing factors in the volitional phase include self-monitoring [26], goal-setting and implementation intentions [27, 28], coping planning (via volitional sheets) [29], and behavioral goal reminders [30, 31]. For the experimental group the WhiteTeeth app is available in the App Store for IOS ≥7+ and in the Play Store for Android ≥4.1 as ‘Witgebit’ (free of charge).

Upon installing and opening the WhiteTeeth app, participants in the intervention group will be required to respond to registration questions and to provide some personal details. These questions will cover the frequency of tooth brushing, the use of fluoride mouth rinse and dental cleaning aids, the length of brushing sessions, and the type of toothbrush. The personal details pertain to outcomes that motivate them to maintain good oral health. Adolescents can select from pre-established outcome-expectancies, such as gum health, fresh breath, plaque removal and whitening. Messages can thus be individualized.

In the next part of the registration process, which is intended to help adolescents identify their dental plaque levels, they will be asked to use the disclosing tablets and to take a selfie of their teeth on which dental plaque is visualized by the disclosing tablet. The participant has to fit the selfie into a fixed window, upon which the app will superimpose a grid. The adolescent will then be asked to register the amount of plaque by clicking the disclosed areas in the grid. The app will interpret the number of clicks (i.e. absence or presence of plaque). Then, on the basis of this plaque assessment and of the answers to the questions on oral-health procedures during the registration phase, the app will provide feedback. If the adolescent has complied with the oral-health recommendations and if dental plague is absent, positive reinforcement will be given. If the adolescent has not complied with these recommendations or if dental plaque is present, personal oral-health advice will be given in short videos and a peer model (i.e. an adolescent with fixed orthodontic appliances) will demonstrate how to clean teeth with fixed appliances. Adolescents will then set a particular goal with regard to tooth-brushing frequency and duration, the use of a proxy brush and/or fluoride mouth rinse (goal-setting). Next, they will be required to formulate when and where they will perform the oral-health behavior (formulating “if-then” goals or an implementation intention). As part of the implementation planning, the app will provide an option for setting reminders for oral-health-behavior tasks. The action plans formulated will be saved on the main page of the app. At the end of the installation process, the app will encourage adolescents to use the brushing timer daily and to monitor their oral-health behavior by registering in the app whether they have performed their tasks.

Every day throughout the 12-week intervention period, push-notifications will be sent instructing adolescents to use the app to enter whether or not they have accomplished their daily dental activities, and to remind them to use the brushing timer when brushing their teeth (at installation, adolescents set the time at which they would like to receive this notification). When adolescents decide to brush, they have the option of turning on the timer. As well as showing the time elapsed during brushing, the timer supports good tooth brushing by showing where and how to brush according to the 5-step method. When adolescents have completed brushing, the app provides positive reinforcement.

Each week, adolescents will be asked via the app to evaluate their dental plaque levels and review their behavioral goals. For this purpose, they will use a disclosing tablet to visualize the dental plaque. They will also be asked to take a selfie of the result and to indicate the visualized dental plaque (following the same procedure as in the registration phase). On the basis of the information both on the selfie – which indicates an increase or decrease in the number of clicks and thus the amount of plaque – and on the activities performed that week, the app concludes whether the adolescent’s goals have been attained. It then compliments the adolescent for using the app, and, if necessary, helps him or her to set new goals or to adapt the existing ones. If adolescents have failed to attain their goals, they can formulate coping plans, i.e., “if-then” plans specifying how they can deal with difficult situations. To establish these, they use volitional sheets, i.e., sheets with pre-established difficult situations and solutions, such as “If I’m too tired to brush my teeth in the evening, then I’ll brush my teeth right after dinner.” These coping plans will be saved on the main page of the app, so adolescents will be reminded of them when they open the app.

If the adolescent does not use the app, it will send personalized text-messages every three days reminding them to use it. These messages will be based on information obtained during installation of the app. For example, “Brushing will help to keep your teeth healthy and beautiful.”

For two weeks, a prototype of the app was pre-tested by 28 adolescents with fixed orthodontic appliances. The data from this pilot test showed that the adolescents appreciated the app for its high usability and were very satisfied with it – particularly with the videos.

### Data collection

Figure 1 presents an overview of the data-collection procedures. Before the orthodontic check-ups at T0, T1 and T2, all participants will fill in a digital questionnaire on the tablet (Ipad® Air 2.0) in a separate room at the clinic. Next, before the orthodontic check-up, the clinical measurements will be made.

#### Demographic or background information

The first part of the self-administered digital questionnaire includes questions on the participants’ demographic background and any possible confounding variables (age, sex, education level, nationality, cultural background, and smoking status (see Additional file 2 for questionnaire part I)). Information will be retrieved from the orthodontic files about the date on which treatment with fixed orthodontic appliances started and about the type of orthodontic bracket (e.g., self-ligating or conventional appliances, and the presence of elastic hooks and/or looped wires).

#### Self-reported oral-health behavior and its psychosocial factors

The second part of the self-administered digital questionnaire contains questions with both single-response and multiple-response items on oral-health behaviors and their psychosocial factors (HAPA factors) (see Additional file 3 for questionnaire part II). This questionnaire was derived from earlier studies on oral health [15, 32–34]. The additional file specifies which questions were derived from which original questionnaire. First, to ensure that the integrity of the content was maintained, the original questions were translated into Dutch and then back-translated to English. Next, to ensure that the questions are comprehensible, the questionnaire was piloted.

The questionnaire asks respondents to report the frequency with which they use a toothbrush, a proxy brush, dental floss, toothpicks, mouth rinse, and other dental aids. It used the following 7-point scale: 1: less than twice a month or never, 2: twice a month, 3: once a week, 4: two to three times weekly, 5: once daily, 6: twice daily, and 7: three times daily or more. For the analysis, these response options will be recalculated to establish the weekly frequencies of each of the oral-health behaviors. Self-reported tooth-brushing frequency and tooth-brushing duration will both be measured in one open question, i.e., “In the last four weeks, how many times have you brushed your teeth per day?” and “How much time do you spend on brushing your teeth at a time?”. For the analysis, these two items will be multiplied to obtain a single item: self-reported tooth-brushing duration (minutes per day).

With regard to the psychosocial factors relevant to tooth brushing and the use of a proxy brush, the questionnaire includes questions on “risk perception”, “action-self-efficacy”, “intention”, “maintenance self-efficacy”, “recovery self-efficacy”, “action control”, “social influences” (including parental support, descriptive and subjective norm), “action planning” and “coping planning”. Next, the questionnaire includes questions on adolescents’ outcome expectancies regarding dental cleaning, and questions about “action-self-efficacy” and “intention” regarding the use of mouth rinse. All psychosocial factors will be assessed on 5-point scales ranging from “very low” (1) to “very high” (5) for risk perception, and from “totally disagree” (1) to “totally agree” (5) for the remaining items. To obtain a single score per psychosocial factor for tooth brushing and the use of a proxy brush, the scores will be summed. The headers in the questionnaire (part II in the Additional file 3) show the items that are summed to generate the score for each psychosocial factor.

#### Clinical measurements

At baseline and at 6 and 12 weeks’ follow-up, the state of adolescents’ oral hygiene will be determined by the amount of plaque and gingival bleeding at the buccal surfaces of the first premolars, canines and incisors. These elements were chosen on the basis of the finding by Chapman et al. (2010) that the maxillary lateral incisor was the tooth most frequently and severely affected by white spot lesions; it was followed by the maxillary canine, premolar, and central incisor [2].

To achieve this, the following clinical assessments will be carried out:

#### Modified Silness and Loë plaque index

A systematic review conducted by Al-Anezi concluded that the modified Silness and Loë plaque index by Williams (1991) is the most valid and discriminatory index for measuring plaque accumulation in orthodontic patients [35, 36]. Using a mouth mirror and a probe, we will therefore use this index to establish the amount of plaque on the buccal surfaces of the first premolars, canines and incisors. According to the position of the orthodontic bracket, the buccal surface of each tooth is divided into four zones, i.e., those mesial, distal, gingival and incisal to the bracket [35, 36]. Each of the four sites of the buccal tooth surface is given a score from 0 to 3, where 0 indicates the absence of plaque; 1 indicates no plaque visible, but an accumulation of soft deposit on a probe when used to clean the surface; 2 indicates a moderate accumulation of soft deposit on the tooth which can be seen with the naked eye; and 3 indicates an abundance of soft matter on the tooth. For the analysis, values are summed to obtain a total score per participant.

#### Bleeding upon marginal probing (BOMP)

Gingival bleeding will be assessed with the Bleeding on Marginal Probing index (BOMP). This will be used to score the condition of the gingiva according to the method described by Van der Weijden et al. (1991). In summary, a periodontal probe (tapered tine, tip diameter 0.5 mm; Hu-Friedy, Liemen, Germany) runs along the soft tissue wall at the orifice of the pocket – i.e., the marginal gingiva – at an angle of approximately 60° to the longitudinal axis of the tooth [37]. To determine whether probing elicited marginal bleeding (score 1) or not (score 0), we will assess the mesio-vestibular, vestibular, disto-vestibular sections of the vestibular surfaces of the first premolar, canines and incisors. The presence or absence of bleeding will be scored within 30 s of probing. To obtain a total number of bleeding sites per participant, all scores for the analysis will be summed.

To ensure the reliability of the clinical measurements, the clinical examiners at each site (a dentist and student dental hygienists) will be trained and calibrated by an experienced ACTA University examiner two weeks before the start of the study. The calibration will involve a separate group of ten people.

It will not be possible to determine inter-rater reliability by performing the clinical measurements twice, as the first set of measurements will affect the results of the second: plaque will removed by the probe used to determine the plaque score, and more marginal bleeding might be elicited by the second probing of marginal gingiva. For this reason, one examiner will perform the clinical measurements and another examiner will observe them, and then give a second independent judgement. The inter-rater reliability will thus be determined by comparing the scores made by both examiners. This will be done in a random sample of 10% of the measurements. To determine the inter-rater reliability of the primary outcome measures the Intra-class Correlation Coefficients (ICC) will be calculated.

#### Process evaluation

A process evaluation will be performed to examine intervention fidelity (i.e., the extent to which participants comply with the intervention); and participants’ experiences with the intervention program, including the perceived effectiveness of various components of the WhiteTeeth app.

Information on intervention fidelity will be collected through the app. For medical-ethical reasons, the app users’ data will not be uploaded automatically. Instead, participants will have to send it themselves from their smartphone to the database. During intervention allocation, participants will be informed about the collection of their users’ data, and will be asked to send it via the app each week. At 6 and 12 weeks follow-up, all participants will be reminded to send their user’s data via the app. User’s data consists of (1) the week that the users’ data is sent, (2) the login code of the app, (3) the total number of selfies, (4) the total number of clicks on the selfie, (5) the total number of times that the brushing timer is opened, (6) the average number of minutes brushed registered by the brushing-timer, (7) the action plans that are entered into the application, (8) the goals that were set, (9) the number of times that they watch a video about (I) dental plaque and cleaning their teeth either with (II) a manual toothbrush, an (III) electric toothbrush, or (IV) interdental brushes; and (10) their initial motives for cleaning their teeth.

At 12 weeks follow-up, the participants’ experiences with the intervention program will be examined in a short questionnaire consisting of open and closed questions (See Additional file 4 for questionnaire part III).

To maximize retention of participants, all participants will receive a letter at baseline on the importance of adhering to the intervention and on participation in the follow-up measurements. To promote their active participation in the study, participants will receive monetary compensation (€10) at the end of the trial.

### Data-management

All data will be recorded using FileMaker Pro© 15 database. Separate FileMaker Pro forms have been designed for both the clinical measurements and the questionnaires.

The researchers will register clinical data on tablets. To fill in all questionnaires, participants will use the tablets in a separate room. To ensure that appropriate help and guidance can be given when needed, this will be done in the presence of one of the researchers. To minimize data-entry errors, the forms have inbuilt check-and-skip rules. FileMaker Pro will allow us to export the data safely from tablets to SPSS Statistics database. For participants who withdraw from the trial, any data collected up to the withdrawal date will be retained and included in the analyses.

Names, mobile phone numbers and addresses will not be recorded in the same forms as sensitive data. Each participant will be given a unique identification number. Each participant will send their weekly user’s data to a secure server owned by ACTA University. Only the principal and the co-principal researchers will have access to the participants’ personal data. All data will be saved on password-protected computers and tablets. When the study has been completed, all personal identifiers will be deleted. Data will be kept in stored digitally at the coordinating center (ACTA) for 5 years after completion of the study.

### Blinding

The participants in this study cannot be blinded for the intervention allocation after randomization. To ensure the blindness of assessors and clinicians, the principal researcher will ask the participants not to communicate with outcome assessors or their clinicians on whether they use the app.

### Sample size

The sample-size calculation will be based on the primary outcome measure, the modified Silness and Loë plaque index. Between treatment groups we have assumed a 0.35 difference in mean change at week 12 [38]. The required sample size is 2 × 63 patients (setting α = 5% (two-tailed), a standard deviation (s) of 0.7, and the power (1-β) = 0.80) [39]. Allowing for an expected loss of 15%, a sample of 73 patients is needed in each group.

### Statistical analysis

All statistical analysis will be based on the intention-to-treat (ITT) principle. The participants’ characteristics will be summarized using descriptive statistics (mean, standard deviation, frequency). Baseline data will be used to investigate the characteristics of any participants who discontinue or deviate from the trial and/or intervention. The magnitude of change over time across study groups will be examined by linear mixed models for continuous primary and secondary outcome variables, controlling for baseline variables and other covariates that may relate to the outcome.

To take account of the correlated observations within the participant, mixed-model analyses will be used. Two analyses will be performed: 1) to evaluate the overall intervention effect, 2) to evaluate the intervention effect at different follow-up times. This will be done by adding time and the interaction between time and intervention group variable into the linear mixed models. For mediation analysis, we will perform linear regressions based on Baron and Kenny’s recommendations [40]. A Sobel test will be used to test the mediating effect. A z-value greater than 1.96 and a *p*-value lower than 0.05 will indicate a significant mediating effect [41].

### Benefits and harms

Participants in the experimental condition might benefit from the intervention by achieving better oral health. Since no harmful consequences are expected from exposure to the intervention, a data-monitoring committee is not needed. Unanticipated problems or adverse events that are likely to be related to the trial will be recorded and reported to the METC at VU Medical Centre Amsterdam. Authorship of the publications emerging from the study will be decided on the basis of the guidelines of the International Committee of Medical Journal Editors.

### Dissemination plan

This protocol was written according to the Standard Protocol Items: Recommendations for Intervention Trials (SPIRIT) guidelines [42] (see Additional files 5 and 6). The research findings will be disseminated through reports, presentations, and scientific articles in peer-reviewed journals. Findings will be reported according to the Consolidated Standards of Reporting Trials (CONSORT) guidelines [43]. Important protocol modifications will be reported when findings are disseminated.

## Discussion

This paper describes the protocol for an RCT to evaluate the effectiveness of the WhiteTeeth app, which is intended to prevent dental caries in adolescents with fixed orthodontic appliances by improving oral-health behavior and oral hygiene more than care as usual does. By making our study objectives and methods known, the publication of this study protocol will improve the eventual usefulness of our study [44].

The WhiteTeeth app intervention was developed systematically on the basis of the Intervention Mapping protocol [19]. This protocol guides the linking of theory to specific behavior-change targets and their associated behavior-change techniques and delivery methods [19]. The WhiteTeeth app is based on the HAPA theory, in which changing health-related behaviors comprises two consecutive behavioral phases that are essential to achieving behavior change: a motivational phase and a volitional phase. As the systematic theoretical underpinnings of WhiteTeeth allow additional questions to be addressed regarding the influence of mediating factors on outcomes, we will be able to increase our understanding of the extent to which the outcomes in dental hygiene can be explained by some or all of the underlying psychosocial and behavioral factors (mediators). The process evaluation will provide additional insight into the effective ingredients of the intervention and into the feasibility of the intervention for the target group. Understanding of these issues will underlie the post-trial adjustments necessary to enhancing the effectiveness of WhiteTeeth before any larger scale roll-out.

We should note some weaknesses in the design of the intervention. First, data on oral-health behaviors and its psychosocial factors will be self-reported. Self-reported measures are prone to bias, such as social desirability bias. As far as possible, we will therefore use the users’ data for several components of the app to evaluate whether the self-reported behavior corresponds to this data. For example, the mean brushing duration collected by the brushing timer of the app will be compared with the self-reported tooth-brushing duration.

A second limitation is that the participants in the control group might also undergo changes in oral-health behavior, which may conceivably be induced by questions about their behavior. Wilding et al. (2016) have shown that participants’ behavior can be increased or changed simply if questions are asked about their behavior [45]. In our study, there is thus a chance that the effects of asking people about their behavior will reduce any differences between the intervention and the control groups, and thereby the possibility of finding a significant effect.

Although the intervention has been developed to prevent dental caries, this specific health outcome will not be measured in this RCT. Dental caries is nonetheless strongly associated with the outcome measures in the present study: oral-health behavior and oral hygiene measures [11, 46]. To prevent caries entirely, good oral-health behavior should be maintained continuously over a long period of time. As habit-formation takes an average of 66 days [47], we expect that the 84 days of exposure to the intervention will be long enough to guarantee a long-term behavior change. We assume that if the WhiteTeeth app is effective in improving adolescents’ oral-health behavior and oral hygiene status, it is also likely to affect caries development.

Thus, if our study confirms the effects we hypothesize on oral-health behavior and oral hygiene status, we will recommend that long-term studies are carried out with dental caries as a primary long-term outcome. On the hypothesis that our preventive oral-health intervention reduces orthodontic patients’ long-term health costs, such long-term follow-up studies should also incorporate health-care costs.

### Trial status

Recruitment started in November 2016 and was continuing when the manuscript was submitted. Inclusion is estimated to finish in October 2017.

## Additional files

Additional file 1: Informed Consent Forms. (DOCX 14 kb)

Additional file 2: Questionnaire part I. (DOCX 22 kb)

Additional file 3: Questionnaire part II. (DOCX 67 kb)

Additional file 4: Questionnaire part III. (DOCX 25 kb)

Additional file 5: Figure SPIRIT Schedule of enrolment, interventions, and assessments. (DOCX 16 kb)

Additional file 6: SPIRIT Checklist. (DOCX 50 kb)

Additional file 7: A copy of the proof of funding. (PDF 292 kb)

Additional file 8: A copy of the proof ethics (METC protocol. nr 2016.162). PDF (42 kb)

## Abbreviations

ACTA: Academic centre for Dentistry Amsterdam
App: Application
BCTs: Behavior Change Techniques
BOMP: Bleeding On Marginal Probing index
CONSORT: Consolidated Standards of Reporting Trials
HAPA: Health Action Process Approach
METC: Medical Ethics Committee
PI: Modified Silness and Loë Plaque Index
RCT: Randomized Controlled Trial
SPIRIT: Standard Protocol Items: Recommendations for Intervention Trials
