# Supplementary material for: Study protocol of a randomized controlled trial to test the effect of a smartphone application on oral-health behavior and oral hygiene in adolescents with fixed orthodontic appliances
Source: BMC Oral Health. 2018 Feb 7;18:19. doi: 10.1186/s12903-018-0475-9 (PMC5803887; doi:10.1186/s12903-018-0475-9)
Supplement: Supplementary file 1 — Informed Consent Forms. (DOCX 14 kb) [file 12903_2018_475_MOESM1_ESM.docx]

Additional file 1:

Informed Consent Forms
 **Participant’s consent form**

**Study: The WhiteTeeth app for improving oral hygiene.**

- I have read the information letter. I had the opportunity to ask questions. If I had any questions, they were answered properly. I was given plenty of time to make a decision on participating in the study.
- I know the purpose of the research and how much time it will take me to participate in it.
- I give my full consent to the use of my data for the express purposes detailed in the information letter. I fully consent to the fact that all data will be stored for up to 5 years after completion of the research.
- I know that participation is voluntary. I know that I can decide at any given moment to withdraw this participation without stating a reason.
- I agree to participate in this research.

Name of participant:

Signature: Date : __ / __ / __

**Parent’s/Guardian’s consent form Study:
The WhiteTeeth app for improving oral hygiene.**

- I have read the information letter. I had the opportunity to ask questions. If I had any questions, they were answered properly. I was given plenty of time to decide on my child’s participation in the study.
- I declare that I have been adequately informed on the nature, purpose and burden of the research.
- I give my full consent to the acquisition and use of my child’s data 1.) through the methods described and 2.) for the express purposes detailed in the information letter. I fully consent to the fact that my child’s data will be stored for up to 5 years after completion of the research.
- I voluntarily consent to my child’s participation in this research. I maintain the right to withdraw this consent without stating a reason.

Name of child: .....................................................................................

Name of parent: .....................................................................................

Signature: Date : __ / __ / __
