# Supplementary material for: Study protocol of a randomized controlled trial to test the effect of a smartphone application on oral-health behavior and oral hygiene in adolescents with fixed orthodontic appliances
Source: BMC Oral Health. 2018 Feb 7;18:19. doi: 10.1186/s12903-018-0475-9 (PMC5803887; doi:10.1186/s12903-018-0475-9)
Supplement: Supplementary file 2 — Questionnaire part I. (DOCX 22 kb) [file 12903_2018_475_MOESM2_ESM.docx]

Additional file 2:

Questionnaire Part I: DEMOGRAPHIC AND BACKGROUND VARIABLES

*We will remove the references and the numbers to indicate the range of the score from the questionnaire when used.*

How to answer the questions in this questionnaire:

1. Answer the questions on your own. Don’t discuss them with others!

2. Answer all questions honestly.

3. There are no right or wrong answers; it’s about what you think or what you do.

| 1 | Are you a boy or a girl? ^1^ | | | | | |
| --- | --- | --- | --- | --- | --- | --- |
|  | 🔾^0^ 🔾^1^ | Boy  Girl | | | | |
|  | | | | | | |
| 2 | How old are you? ^1^ | | ….. | years | | |
|  | | | | | | |
| 3 | What level of education are you attending right now? ^1^ | | | | | |
|  | 🔾^1^ 🔾^2^ 🔾^3^ 🔾^4^ 🔾^5^  🔾 | Primary education  Practical pathway or pre-vocational education (PP VMBO)  Theoretical pathway or pre-vocational education (TP VMBO)  Senior general secondary education (HAVO)  Pre-university education (VWO)  Other: ………..……………… | | | | |
|  | | | | | | |
| 4 | To which culture do you feel you belong? ^2^ | | | | | |
|  | 🔾^1^ 🔾^2^ 🔾^3^ 🔾^4^ 🔾^5^ | Dutch  Turkish  Moroccan Surinames  Other culture (please fill in below):  ………………… | | | | |
|  | | | | | | |
| 5 | Do you smoke? ^1^ | | | | | |
|  | 🔾^1^ 🔾^0^ | Yes  No | | | | |
|  | | | | | | |
| 6 | What kind of toothbrush do you use to brush your teeth? | | | | | |
|  | 🔾^1^  🔾^2^ 🔾^3^ | Manual toothbrush Electric toothbrush  Manual toothbrush and electric toothbrush | | | | |
|  | | | | | | |
| 7 | To measure how often between meals your teeth are exposed to the acids or sugars in foods and/or drinks, we would like you to count the number of times you eat or drink between meals in an average day (for example yesterday). If you eat and drink at the same time, it counts as 1 time. If there is more than half an hour between the eating or drinking, it counts as 2 times.  Please tell us the number of times you drink and/or eat between the main meals. Drinks include lemonade, iced tea, energy drinks or orange juice. Do not count water, coffee or tea without sugar. Sugar-free chewing gum does not count as eating. But please count coffee or tea with sugar.  How often do you drink and/or eat anything in between your main meals on an average day? | | | | ……… | per day |

***References****:*

^1^ *RIVM (2005) Lokale en Nationale Monitor gezondheid;* [*https://www.monitorgezondheid.nl/jeugdindicatoren.aspx*](https://www.monitorgezondheid.nl/jeugdindicatoren.aspx) *(accessed september 2016).*

^2^ *Schuller, A., van Kempen, I., Vermaire, E., Poorterman, J., Verlinden, A., Hofstetter, H., & Verrips, E. Een onderzoek naar de mondgezondheid en het tandheelkundig preventief gedrag van volwassenen in Nederland in 2013. Leiden: TNO; 2014.*
