# Supplementary material for: Study protocol of a randomized controlled trial to test the effect of a smartphone application on oral-health behavior and oral hygiene in adolescents with fixed orthodontic appliances
Source: BMC Oral Health. 2018 Feb 7;18:19. doi: 10.1186/s12903-018-0475-9 (PMC5803887; doi:10.1186/s12903-018-0475-9)
Supplement: Supplementary file 3 — Questionnaire part II. (DOCX 67 kb) [file 12903_2018_475_MOESM3_ESM.docx]

Additional file 3:
QUESTIONNAIRE PART II:
ORAL HEALTH BEHAVIOR & ITS PSYCHOSOCIAL FACTORS

*The blue headers in the questionnaire indicate what constructs will be measured. We will remove the references, the numbers to indicate the range of the score and the blue headers from the questionnaire when used.*

*Abbreviations: TB = regarding Tooth Brushing; PB = regarding Proxy Brush; MR = regarding Mouth Rinse; DC = regarding Dental Cleaning, q=question.*

| ORAL HEALTH BEHAVIOUR (q1-5); TOOTH-BRUSHING (q6, q1/q7/q10),  PROX-BRUSH USE (q2/q8/q11) AND FLUORIDE MOUTH RINSE USE (q4/q9/q12) ^[1,2]^ | | | | | | | | | | | | | | | | | | | | | | | | | | | | | |
| --- | --- | --- | --- | --- | --- | --- | --- | --- | --- | --- | --- | --- | --- | --- | --- | --- | --- | --- | --- | --- | --- | --- | --- | --- | --- | --- | --- | --- | --- |
|  | | | | | | | | | | | | | | | | | | | | | | | | | | | | | |
| How many times in the last 4 weeks have you used the products or dental aids listed below? | | | | | | | | | | | | | | | | | | | | | | | | | | | | | |
|  | |  | 3 times per day or more | | Twice a day | | | | | Once a day | | | | | 2 to 3 times a week | | | | Once a week | | | | Twice a month | | | | | Less often or never | |
| 1 | | Toothbrush | 🔾^24.5^ | | 🔾^14^ | | | | | 🔾^7^ | | | | | 🔾^2.5^ | | | | 🔾^1^ | | | | 🔾^.45^ | | | | | 🔾^0^ | |
| 2 | | Proxy brush  (also known as interdental brush/small brush) | 🔾^24.5^ | | 🔾^14^ | | | | | 🔾^7^ | | | | | 🔾^2.5^ | | | | 🔾^1^ | | | | 🔾^.45^ | | | | | 🔾^0^ | |
| 3 | | Toothpick | 🔾^24.5^ | | 🔾^14^ | | | | | 🔾^7^ | | | | | 🔾^2.5^ | | | | 🔾^1^ | | | | 🔾^.45^ | | | | | 🔾^0^ | |
| 4 | | Fluoride mouth rinse | 🔾^24.5^ | | 🔾^14^ | | | | | 🔾^7^ | | | | | 🔾^2.5^ | | | | 🔾^1^ | | | | 🔾^.45^ | | | | | 🔾^0^ | |
| 5 | | Other dental aids, (please specify) ………….……….. (e.g., Dental floss) | 🔾^24.5^ | | 🔾^14^ | | | | | 🔾^7^ | | | | | 🔾^2.5^ | | | | 🔾^1^ | | | | 🔾^.45^ | | | | | 🔾^0^ | |
|  | | | | | | | | | | | | | | | | | | | | | | | | | | | | | |
| Please fill in your tooth-brushing duration, e.g., 2.5 minutes. | | | | | | | | | | | | | | | | | | | | | | | | | | | | | |
| 6 | | Each time you brush your teeth, how much time do you spend on brushing? | | | | | | | | | | | | | | | | | | | ……… | | | | Minutes per brushing | | | | |
|  | |  |  |  | | |  | | | | | | | |  | | | | |  | | | |  | | | | |  |
| How often did you… | | | | | | | | | | | | | | | 3 times or more | | | | | Twice | | | | Once | | | | | I didn’t do this |
| 7 | | brush your teeth yesterday? | | | | | | | | | | | | | 🔾^3^ | | | | | 🔾^2^ | | | | 🔾^1^ | | | | | 🔾^0^ |
| 8 | | use the proxy brush yesterday? | | | | | | | | | | | | | 🔾^3^ | | | | | 🔾^2^ | | | | 🔾^1^ | | | | | 🔾^0^ |
| 9 | | use fluoride mouth rinse yesterday? | | | | | | | | | | | | | 🔾^3^ | | | | | 🔾^2^ | | | | 🔾^1^ | | | | | 🔾^0^ |
|  | |  |  |  | | |  | | | | | | | |  | | | | |  | | | |  | | | | |  |
| In the last 4 weeks, how many times have you… | | | | | | | | | | | | | | | | | | | | | | | | | | | | | |
| 10 | | brushed your teeth? | | | | | | | | | | | | | | | | | | | | ……… | | | | per day | | | |
| 11 | | used the proxy brush? | | | | | | | | | | | | | | | | | | | | ……… | | | | per day | | | |
| 12 | | used fluoride mouth rinse? | | | | | | | | | | | | | | | | | | | | ……… | | | | per day | | | |
|  | |  |  |  | | |  | | | | | | | |  | | | | |  | | | |  | | | | |  |
| If you use a proxy brush regularly, please answer the question below. If not, skip this question*. (This will be done automatically in the digital questionnaire.)* | | | | | | | | | | | | | | | | | | | | | | | | | | | | | |
| Do you use the proxy brush to clean the space… | | | | | | | | | | | | | | | Yes | | | | | | | | No | | | | | | |
| 11b | in between the teeth? | | | | | | | | | | | | | | 🔾^1^ | | | | | | | | 🔾^0^ | | | | | | |
| 11c | in between the brackets? | | | | | | | | | | | | | | 🔾^1^ | | | | | | | | 🔾^0^ | | | | | | |
|  | |  |  |  | | |  | | | | | | | |  | | | | |  | | | |  | | | | |  |
|  | |  |  |  | | |  | | | | | | | |  | | | | |  | | | |  | | | | |  |
| INTENTION TB (q13) & PB (q14a) & MR (q14b) ^[2]^ | | | | | | | | | | | | | | | | | | | | | | | | | | | | | |
|  | |  |  |  | | |  | | | | | | | |  | | | | |  | | | |  | | | | |  |
| How true are the following statements? | | | | | | | | | | | | | | | | | | | | | | | | | | | | | |
| In the next 4 weeks I intend to… | | | | | | Totally untrue | | | | | | Untrue | | | | | Maybe true, maybe untrue | | | | | | True | | | | Totally True | | |
| 13a | | brush my teeth daily at least twice a day. | | | | 🔾^1^ | | | | | | 🔾^2^ | | | | | 🔾^3^ | | | | | | 🔾^4^ | | | | 🔾^5^ | | |
| 13b | | brush my teeth daily for at least 3 minutes each time. | | | | 🔾^1^ | | | | | | 🔾^2^ | | | | | 🔾^3^ | | | | | | 🔾^4^ | | | | 🔾^5^ | | |
| 14a | | use the proxy brush to clean my teeth daily. | | | | 🔾^1^ | | | | | | 🔾^2^ | | | | | 🔾^3^ | | | | | | 🔾^4^ | | | | 🔾^5^ | | |
| 14b | | use fluoride mouth rinse daily. | | | | 🔾^1^ | | | | | | 🔾^2^ | | | | | 🔾^3^ | | | | | | 🔾^4^ | | | | 🔾^5^ | | |
|  | |  |  |  | | |  | | | | | | | |  | | | | |  | | | |  | | | | |  |
| (TASK/) ACTION SELF-EFFICACY TB (q15-18) & PB (q19-21) & MR (q21b) ^[3]^ | | | | | | | | | | | | | | | | | | | | | | | | | | | | | |
|  | |  |  |  | | |  | | | | | | | |  | | | | |  | | | |  | | | | |  |
| How true are the following statements? | | | | | | | | | | | | | | | | | | | | | | | | | | | | | |
| I am confident that I can… | | | | | | | | | | | Totally disagree | | | | Disagree | | | | | Don’t agree, don’t disagree | | | | Agree | | | | | Totally agree |
| 15 | | brush my teeth daily for at least 3 minutes. | | | | | | | | | 🔾^1^ | | | | 🔾^2^ | | | | | 🔾^3^ | | | | 🔾^4^ | | | | | 🔾^5^ |
| 16 | | brush my teeth at least twice a day. | | | | | | | | | 🔾^1^ | | | | 🔾^2^ | | | | | 🔾^3^ | | | | 🔾^4^ | | | | | 🔾^5^ |
| 17 | | attentively brush my teeth daily. | | | | | | | | | 🔾^1^ | | | | 🔾^2^ | | | | | 🔾^3^ | | | | 🔾^4^ | | | | | 🔾^5^ |
| 18 | | brush my teeth daily, even the surfaces that are hard to reach. | | | | | | | | | 🔾^1^ | | | | 🔾^2^ | | | | | 🔾^3^ | | | | 🔾^4^ | | | | | 🔾^5^ |
| 19 | | use the proxy brush daily. | | | | | | | | | 🔾^1^ | | | | 🔾^2^ | | | | | 🔾^3^ | | | | 🔾^4^ | | | | | 🔾^5^ |
| 20 | | attentively clean my teeth with a proxy brush daily. | | | | | | | | | 🔾^1^ | | | | 🔾^2^ | | | | | 🔾^3^ | | | | 🔾^4^ | | | | | 🔾^5^ |
| 21 | | clean my teeth with a proxy brush daily, even the surfaces that are hard to reach. | | | | | | | | | 🔾^1^ | | | | 🔾^2^ | | | | | 🔾^3^ | | | | 🔾^4^ | | | | | 🔾^5^ |
| 21b | | use fluoride mouth rinse daily. | | | | | | | | | 🔾^1^ | | | | 🔾^2^ | | | | | 🔾^3^ | | | | 🔾^4^ | | | | | 🔾^5^ |
|  | |  |  |  | | |  | | | | | | | |  | | | | |  | | | |  | | | | |  |
|  | |  |  |  | | |  | | | | | | | |  | | | | |  | | | |  | | | | |  |
| MAINTENANCE SELF-EFFICACY TB (q22-25) ^[3]^ | | | | | | | | | | | | | | | | | | | | | | | | | | | | | |
|  | |  |  |  | | |  | | | | | | | |  | | | | |  | | | |  | | | | |  |
| I am confident that I can brush my teeth for 3 minutes at least twice a day… | | | | | | | | | | | Totally disagree | | | | Disagree | | | | | Don’t agree, don’t disagree | | | | Agree | | | | | Totally agree |
| 22 | | even when I do not see immediate results. | | | | | | | | | 🔾^1^ | | | | 🔾^2^ | | | | | 🔾^3^ | | | | 🔾^4^ | | | | | 🔾^5^ |
| 23 | | even when I don’t feel like doing it. | | | | | | | | | 🔾^1^ | | | | 🔾^2^ | | | | | 🔾^3^ | | | | 🔾^4^ | | | | | 🔾^5^ |
| 24 | | even when I’m in a hurry. | | | | | | | | | 🔾^1^ | | | | 🔾^2^ | | | | | 🔾^3^ | | | | 🔾^4^ | | | | | 🔾^5^ |
| 25 | | even if it takes a lot of time. | | | | | | | | | 🔾^1^ | | | | 🔾^2^ | | | | | 🔾^3^ | | | | 🔾^4^ | | | | | 🔾^5^ |
|  | |  |  |  | | |  | | | | | | | |  | | | | |  | | | |  | | | | |  |
| MAINTENANCE SELF-EFFICACY & PB (q26-29) ^[3]^ | | | | | | | | | | | | | | | | | | | | | | | | | | | | | |
|  | |  |  |  | | |  | | | | | | | |  | | | | |  | | | |  | | | | |  |
| I am confident that I can clean my teeth with a proxy brush daily… | | | | | | | | | | | Totally disagree | | | | Disagree | | | | | Don’t agree, don’t disagree | | | | Agree | | | | | Totally agree |
| 26 | | even when I do not see immediate results. | | | | | | | | | 🔾^1^ | | | | 🔾^2^ | | | | | 🔾^3^ | | | | 🔾^4^ | | | | | 🔾^5^ |
| 27 | | even when I don’t feel like doing it. | | | | | | | | | 🔾^1^ | | | | 🔾^2^ | | | | | 🔾^3^ | | | | 🔾^4^ | | | | | 🔾^5^ |
| 28 | | even when I’m in a hurry. | | | | | | | | | 🔾^1^ | | | | 🔾^2^ | | | | | 🔾^3^ | | | | 🔾^4^ | | | | | 🔾^5^ |
| 29 | | even if it takes a lot of time. | | | | | | | | | 🔾^1^ | | | | 🔾^2^ | | | | | 🔾^3^ | | | | 🔾^4^ | | | | | 🔾^5^ |
|  | |  |  |  | | |  | | | | | | | |  | | | | |  | | | |  | | | | |  |
| RECOVERY SELF-EFFICACY TB (q30) & PB (q31) ^[3]^ | | | | | | | | | | | | | | | | | | | | | | | | | | | | | |
|  | |  |  |  | | |  | | | | | | | |  | | | | |  | | | |  | | | | |  |
| Even in the long term, I’m confident that I can… | | | | | | | | | | Totally disagree | | | | | Disagree | | | | | Neither agree nor disagree | | | | Agree | | | | | Totally agree |
| 30a | | brush my teeth at least twice a day. | | | | | | | | 🔾^1^ | | | | | 🔾^2^ | | | | | 🔾^3^ | | | | 🔾^4^ | | | | | 🔾^5^ |
| 30b | | brush my teeth daily for at least 3 minutes at a time. | | | | | | | | 🔾^1^ | | | | | 🔾^2^ | | | | | 🔾^3^ | | | | 🔾^4^ | | | | | 🔾^5^ |
| 31 | | use the proxy brush to clean my teeth daily. | | | | | | | | 🔾^1^ | | | | | 🔾^2^ | | | | | 🔾^3^ | | | | 🔾^4^ | | | | | 🔾^5^ |
|  | |  |  |  | | |  | | | | | | | |  | | | | |  | | | |  | | | | |  |
|  | |  |  |  | | |  | | | | | | | |  | | | | |  | | | |  | | | | |  |
| ACTION PLANNING TB (q32-34) & PB (q35-37) ^[2]^ | | | | | | | | | | | | | | | | | | | | | | | | | | | | | |
|  | |  |  |  | | |  | | | | | | | |  | | | | |  | | | |  | | | | |  |
| Please answer the following questions. | | | | | | | | | | | | | | | | | | | | | | | | | | | | | |
| Do you have a clear plan for… | | | | | | | | | | | | | | | No plan | | | | | Vague plan | | | | Clear plan | | | | | Very clear plan |
| 32 | | when to brush your teeth? | | | | | | | | | | | | | 🔾^1^ | | | | | 🔾^2^ | | | | 🔾^3^ | | | | | 🔾^4^ |
| 33 | | where to brush your teeth? | | | | | | | | | | | | | 🔾^1^ | | | | | 🔾^2^ | | | | 🔾^3^ | | | | | 🔾^4^ |
| 34 | | how much time to spend on brushing your teeth? | | | | | | | | | | | | | 🔾^1^ | | | | | 🔾^2^ | | | | 🔾^3^ | | | | | 🔾^4^ |
| 35 | | when to clean your teeth with a proxy brush? | | | | | | | | | | | | | 🔾^1^ | | | | | 🔾^2^ | | | | 🔾^3^ | | | | | 🔾^4^ |
| 36 | | where to clean your teeth with a proxy brush? | | | | | | | | | | | | | 🔾^1^ | | | | | 🔾^2^ | | | | 🔾^3^ | | | | | 🔾^4^ |
| 37 | | how much time to spend on cleaning your teeth with a proxy brush? | | | | | | | | | | | | | 🔾^1^ | | | | | 🔾^2^ | | | | 🔾^3^ | | | | | 🔾^4^ |
|  | |  |  |  | | |  | | | | | | | |  | | | | |  | | | |  | | | | |  |
| COPING PLANNING TB (q38-39, q42) & PB (q40-41, q43) ^[2]^ | | | | | | | | | | | | | | | | | | | | | | | | | | | | | |
|  | |  |  |  | | |  | | | | | | | |  | | | | |  | | | |  | | | | |  |
| Do you have a clear plan for… | | | | | | | | | | | | | | | No plan | | | | | Vague plan | | | | Clear plan | | | | | Very clear plan |
| 38 | | something hinders brushing? | | | | | | | | | | | | | 🔾^1^ | | | | | 🔾^2^ | | | | 🔾^3^ | | | | | 🔾^4^ |
| 39 | | you forget to brush your teeth? | | | | | | | | | | | | | 🔾^1^ | | | | | 🔾^2^ | | | | 🔾^3^ | | | | | 🔾^4^ |
| 40 | | something hinders using the proxy brush? | | | | | | | | | | | | | 🔾^1^ | | | | | 🔾^2^ | | | | 🔾^3^ | | | | | 🔾^4^ |
| 41 | | you forget to clean your teeth with a proxy brush? | | | | | | | | | | | | | 🔾^1^ | | | | | 🔾^2^ | | | | 🔾^3^ | | | | | 🔾^4^ |
|  | |  |  |  | | |  | | | | | | | |  | | | | |  | | | |  | | | | |  |
| Do you have a plan on how to motivate yourself when you don’t feel like… | | | | | | | | | | | | | | | No plan | | | | | Vague plan | | | | Clear plan | | | | | Very clear plan |
| 42 | | brushing? | | | | | | | | | | | | | 🔾^1^ | | | | | 🔾^2^ | | | | 🔾^3^ | | | | | 🔾^4^ |
| 43 | | using the proxy brush? | | | | | | | | | | | | | 🔾^1^ | | | | | 🔾^2^ | | | | 🔾^3^ | | | | | 🔾^4^ |
|  | |  |  |  | | |  | | | | | | | |  | | | | |  | | | |  | | | | |  |
|  | | | | | | | | | | | | | | | | | | | | | | | | | | | | | |
| OUTCOME EXPECTATIONS DC (q44-49) ^[1]^ | | | | | | | | | | | | | | | | | | | | | | | | | | | | | |
|  | |  |  |  | | |  | | | | | | | |  | | | | |  | | | |  | | | | |  |
| By cleaning my teeth regularly… | | | | | | | Totally disagree | | | | | | | Disagree | | | | Neither agree nor disagree | | | | | Agree | | | | | | Totally agree |
| 44 | | I avoid getting cavities. | | | | | 🔾^1^ | | | | | | | 🔾^2^ | | | | 🔾^3^ | | | | | 🔾^4^ | | | | | | 🔾^5^ |
| 45 | | my breath is fresh. | | | | | 🔾^1^ | | | | | | | 🔾^2^ | | | | 🔾^3^ | | | | | 🔾^4^ | | | | | | 🔾^5^ |
| 46 | | I avoid discoloration of my teeth. | | | | | 🔾^1^ | | | | | | | 🔾^2^ | | | | 🔾^3^ | | | | | 🔾^4^ | | | | | | 🔾^5^ |
| 47 | | I feel fresh. | | | | | 🔾^1^ | | | | | | | 🔾^2^ | | | | 🔾^3^ | | | | | 🔾^4^ | | | | | | 🔾^5^ |
| 48 | | I keep my gums healthy. | | | | | 🔾^1^ | | | | | | | 🔾^2^ | | | | 🔾^3^ | | | | | 🔾^4^ | | | | | | 🔾^5^ |
| 49 | | I look better. | | | | | 🔾^1^ | | | | | | | 🔾^2^ | | | | 🔾^3^ | | | | | 🔾^4^ | | | | | | 🔾^5^ |
|  | |  |  |  | | |  | | | | | | | |  | | | | |  | | | |  | | | | |  |
| RISK PERCEPTION TOWARDS ORAL DISEASES (q50-53), TB (q54-56) & PB (q57-59) ^[3]^ | | | | | | | | | | | | | | | | | | | | | | | | | | | | | |
|  | |  |  |  | | |  | | | | | | | |  | | | | |  | | | |  | | | | |  |
| What is your risk of getting… | | | | | | | Very low | | | | | | | | Low | | | | | Not low, not high | | | | High | | | | | Very high |
| 50. | | a cavity? | | | | | 🔾^1^ | | | | | | | | 🔾^2^ | | | | | 🔾^3^ | | | | 🔾^4^ | | | | | 🔾^5^ |
| 51. | | gum inflammation? | | | | | 🔾^1^ | | | | | | | | 🔾^2^ | | | | | 🔾^3^ | | | | 🔾^4^ | | | | | 🔾^5^ |
|  | |  |  |  | | |  | | | | | | | |  | | | | |  | | | |  | | | | |  |
| Compared to my friends, my risk of getting… | | | | | | | Very low | | | | | | | | Low | | | | | Not low, not high | | | | High | | | | | Very high |
| 52. | | a cavity is: | | | | | 🔾^1^ | | | | | | | | 🔾^2^ | | | | | 🔾^3^ | | | | 🔾^4^ | | | | | 🔾^5^ |
| 53. | | a gum inflammation is: | | | | | 🔾^1^ | | | | | | | | 🔾^2^ | | | | | 🔾^3^ | | | | 🔾^4^ | | | | | 🔾^5^ |
|  | |  |  |  | | |  | | | | | | | |  | | | | |  | | | |  | | | | |  |
| If I don’t brush my teeth often, the risk of getting… | | | | | | | | | Very low | | | | | | Low | | | | | Not low, not high | | | | High | | | | | Very high |
| 54 | | a cavity will be: | | | | | | | 🔾^1^ | | | | | | 🔾^2^ | | | | | 🔾^3^ | | | | 🔾^4^ | | | | | 🔾^5^ |
| 55 | | a gum inflammation will be: | | | | | | | 🔾^1^ | | | | | | 🔾^2^ | | | | | 🔾^3^ | | | | 🔾^4^ | | | | | 🔾^5^ |
| 56 | | discoloration of my teeth will be: | | | | | | | 🔾^1^ | | | | | | 🔾^2^ | | | | | 🔾^3^ | | | | 🔾^4^ | | | | | 🔾^5^ |
|  | |  |  |  | | | | | | | |  | | | |  | | | |  | | | |  | | | | |  |
|  | |  |  |  | | | | | | | |  | | | |  | | | |  | | | |  | | | | |  |
| If I don’t clean my teeth daily with a proxy brush, the risk of getting: | | | | | | | | | Very low | | | | | | | Low | | | | Not low, not high | | | | High | | | | | Very high |
| 57 | | a cavity will be: | | | | | | | 🔾^1^ | | | | | | | 🔾^2^ | | | | 🔾^3^ | | | | 🔾^4^ | | | | | 🔾^5^ |
| 58 | | a gum inflammation will be: | | | | | | | 🔾^1^ | | | | | | | 🔾^2^ | | | | 🔾^3^ | | | | 🔾^4^ | | | | | 🔾^5^ |
| 59 | | discoloration of my teeth will be: | | | | | | | 🔾^1^ | | | | | | | 🔾^2^ | | | | 🔾^3^ | | | | 🔾^4^ | | | | | 🔾^5^ |
|  | |  |  |  | | | | | | | |  | | | |  | | | |  | | | |  | | | | |  |
| SOCIAL INFLUENCES DC (PARENTAL SUPPORT (q60) & DESCRIPTIVE NORM (q61-63)  SUBJECTIVE NORM (q64-66) ^[1]^ | | | | | | | | | | | | | | | | | | | | | | | | | | | | | |
|  | |  |  |  | | |  | | | | | | | |  | | | | |  | | | |  | | | | |  |
| My parents… | | | | | | | Totally disagree | | | | | | Disagree | | | | | Neither agree nor disagree | | | | | | Agree | | | | | Totally agree |
| 60 | | often remind me that I need to clean my teeth properly. | | | | | 🔾^1^ | | | | | | 🔾^2^ | | | | | 🔾^3^ | | | | | | 🔾^4^ | | | | | 🔾^5^ |
| 61 | | brush their teeth at least twice a day. | | | | | 🔾^1^ | | | | | | 🔾^2^ | | | | | 🔾^3^ | | | | | | 🔾^4^ | | | | | 🔾^5^ |
|  | |  |  |  | | |  | | | | | | | |  | | | | |  | | | |  | | | | |  |
| I think that other young people with fixed braces… | | | | | | | Totally disagree | | | | | | | Disagree | | | | Neither agree nor disagree | | | | | | Agree | | | | | Totally agree |
| 62 | | brush their teeth for 3 minutes at least twice a day. | | | | | 🔾^1^ | | | | | | | 🔾^2^ | | | | 🔾^3^ | | | | | | 🔾^4^ | | | | | 🔾^5^ |
| 63 | | use the proxy brush to clean their teeth daily. | | | | | 🔾^1^ | | | | | | | 🔾^2^ | | | | 🔾^3^ | | | | | | 🔾^4^ | | | | | 🔾^5^ |
|  | |  |  |  | | |  | | | | | | | |  | | | | |  | | | |  | | | | |  |
| The following people think that I should clean my teeth properly: | | | | | | | Totally disagree | | | | | | | Disagree | | | | Neither agree nor disagree | | | | | | Agree | | | | | Totally agree |
| 64 | | My friends | | | | | 🔾^1^ | | | | | | | 🔾^2^ | | | | 🔾^3^ | | | | | | 🔾^4^ | | | | | 🔾^5^ |
| 65 | | My dental healthcare provider. | | | | | 🔾^1^ | | | | | | | 🔾^2^ | | | | 🔾^3^ | | | | | | 🔾^4^ | | | | | 🔾^5^ |
| 66 | | My parents or guardian. | | | | | 🔾^1^ | | | | | | | 🔾^2^ | | | | 🔾^3^ | | | | | | 🔾^4^ | | | | | 🔾^5^ |
|  | |  |  |  | | |  | | | | | | | |  | | | | |  | | | |  | | | | |  |
|  | |  |  |  | | |  | | | | | | | |  | | | | |  | | | |  | | | | |  |
| ACTION CONTROL TB (q67-68, q70) & PB (q69, q71) ^[3]^ | | | | | | | | | | | | | | | | | | | | | | | | | | | | | |
|  | |  |  |  | | |  | | | | | | | |  | | | | |  | | | |  | | | | |  |
| In the last week … | | | | | | | | Totally disagree | | | | | | Disagree | | | | Neither agree nor disagree | | | | | Agree | | | | | Totally agree | |
| 67 | | I have regularly checked how often I have brushed my teeth. | | | | | | 🔾^1^ | | | | | | 🔾^2^ | | | | 🔾^3^ | | | | | 🔾^4^ | | | | | 🔾^5^ | |
| 68 | | I have regularly checked my daily tooth-brushing duration. | | | | | | 🔾^1^ | | | | | | 🔾^2^ | | | | 🔾^3^ | | | | | 🔾^4^ | | | | | 🔾^5^ | |
| 69 | | I have regularly checked how often I used the proxy brush. | | | | | | 🔾^1^ | | | | | | 🔾^2^ | | | | 🔾^3^ | | | | | 🔾^4^ | | | | | 🔾^5^ | |
| 70a | | I have really tried hard to brush my teeth twice a day. | | | | | | 🔾^1^ | | | | | | 🔾^2^ | | | | 🔾^3^ | | | | | 🔾^4^ | | | | | 🔾^5^ | |
| 70b | | I have really tried hard to brush my teeth for at least 3 minutes at a time. | | | | | | 🔾^1^ | | | | | | 🔾^2^ | | | | 🔾^3^ | | | | | 🔾^4^ | | | | | 🔾^5^ | |
| 71 | | I have really tried hard to clean my teeth with a proxy brush every day. | | | | | | 🔾^1^ | | | | | | 🔾^2^ | | | | 🔾^3^ | | | | | 🔾^4^ | | | | | 🔾^5^ | |

This is the end of the questionnaire. Thanks for your collaboration!

***References****:*

^1^ *Tolvanen M, Lahti S, Miettunen J, Hausen H. Relationship between oral health-related knowledge, attitudes and behavior among 15–16-year-old adolescents—A structural equation modeling approach. Acta Odontol Scand 2012;70:169-76.*

^2^ *Pakpour AH, Hidarnia A, Hajizadeh E, Plotnikoff RC. Action and coping planning with regard to dental brushing among Iranian adolescents. Psychol Health Med 2012;17:176-87.*

^3^ *Schwarzer R, Schüz B, Ziegelmann JP, Lippke S, Luszczynska A, Scholz U. Adoption and maintenance of four health behaviors: Theory-guided longitudinal studies on dental flossing, seat belt use, dietary behavior, and physical activity. Ann Behav Med 2007;33:156-66.*
