# Supplementary material for: Study protocol of a randomized controlled trial to test the effect of a smartphone application on oral-health behavior and oral hygiene in adolescents with fixed orthodontic appliances
Source: BMC Oral Health. 2018 Feb 7;18:19. doi: 10.1186/s12903-018-0475-9 (PMC5803887; doi:10.1186/s12903-018-0475-9)
Supplement: Supplementary file 4 — Questionnaire part III. (DOCX 25 kb) [file 12903_2018_475_MOESM4_ESM.docx]

Additional file 4:
QUESTIONNAIRE PART III: PARTICIPANTS’ EXPERIENCES WITH THE WHITETEETH APP

*The blue headers in the questionnaire indicate the constructs that are measured. The blue headers, the references, and the scores of the answer options are removed from the questionnaire when used.*

Moet onderstaande niet bij elke vragenlijst?

How to answer the questions in this questionnaire:

1. Answer the questions on your own. Don’t discuss them with others!

2. Answer all questions honestly.

3. There are no right or wrong answers; it’s about what you think or what you do.

|  | | | | | | | | | | | | | | | | | | | | | | | | |
| --- | --- | --- | --- | --- | --- | --- | --- | --- | --- | --- | --- | --- | --- | --- | --- | --- | --- | --- | --- | --- | --- | --- | --- | --- |
| ACCEPTABILITY (q1,15) OPERABILITY (q2-3), ATTRACTIVENESS (q4, q21-23) OF THE APP; USERS’ PERCEPTIONS OF SEVERAL COMPONENTS OF THE APP (q5-14); INTENTION TO USE THE APP (q16); PERCEIVED EFFECTIVENESS (q17); USABILITY (q20, q26-34) - questions are adapted from the system usability scale (SUS) ^[1,2,3,4].^ | | | | | | | | | | | | | | | | | | | | | | | | |
|  | | | | | | | | | | | | | | | | | | | | | | | | |
| **The following questions concern your opinion of the WhiteTeeth app. Indicate per statement whether you think it’s good or bad.** | | | | | | | | | | | | | | | | | | | | | | | | |
| What did you think about…. | | | | | Very bad | | Bad | | | | Neither good, nor bad | | | | Good | | | | Very good | | | | Not applicable | |
| 1 | **The app in its entirety?** | | | | 🔾^1^ | | 🔾^2^ | | | | 🔾^3^ | | | | 🔾^4^ | | | | 🔾^5^ | | | | 🔾 | |
| 2 | **The app’s loading time when you opened it?** | | | | 🔾^1^ | | 🔾^2^ | | | | 🔾^3^ | | | | 🔾^4^ | | | | 🔾^5^ | | | | 🔾 | |
| 3 | **The app’s speed when it was opened?** | | | | 🔾^1^ | | 🔾^2^ | | | | 🔾^3^ | | | | 🔾^4^ | | | | 🔾^5^ | | | | 🔾 | |
| 4 | **The app’s layout?** | | | | 🔾^1^ | | 🔾^2^ | | | | 🔾^3^ | | | | 🔾^4^ | | | | 🔾^5^ | | | | 🔾 | |
| 5 | **The brushing timer?** | | | | 🔾^1^ | | 🔾^2^ | | | | 🔾^3^ | | | | 🔾^4^ | | | | 🔾^5^ | | | | 🔾 | |
| 6 | **The goal-setting option?** | | | | 🔾^1^ | | 🔾^2^ | | | | 🔾^3^ | | | | 🔾^4^ | | | | 🔾^5^ | | | | 🔾 | |
| 7 | **The tutorial at the start of the app?** | | | | 🔾^1^ | | 🔾^2^ | | | | 🔾^3^ | | | | 🔾^4^ | | | | 🔾^5^ | | | | 🔾 | |
| 8 | **The option for setting reminders for your tasks?** | | | | 🔾^1^ | | 🔾^2^ | | | | 🔾^3^ | | | | 🔾^4^ | | | | 🔾^5^ | | | | 🔾 | |
| 9 | **The reminders you received that reminded you of your tasks (pop-ups/notifications on your smartphone)?** | | | | 🔾^1^ | | 🔾^2^ | | | | 🔾^3^ | | | | 🔾^4^ | | | | 🔾^5^ | | | | 🔾 | |
| 10 | **The pop-ups/notifications you received that reminded you to check off your tasks?** | | | | 🔾^1^ | | 🔾^2^ | | | | 🔾^3^ | | | | 🔾^4^ | | | | 🔾^5^ | | | | 🔾 | |
| 11 | **The option for monitoring your progress using the “statistics screen” in the app?** | | | | 🔾^1^ | | 🔾^2^ | | | | 🔾^3^ | | | | 🔾^4^ | | | | 🔾^5^ | | | | 🔾 | |
| 12 | **The duration of the brushing timer?** | | | | 🔾^1^ | | 🔾^2^ | | | | 🔾^3^ | | | | 🔾^4^ | | | | 🔾^5^ | | | | 🔾 | |
| 13 | **The overview of the selfies?** | | | | 🔾^1^ | | 🔾^2^ | | | | 🔾^3^ | | | | 🔾^4^ | | | | 🔾^5^ | | | | 🔾 | |
| 14 | **The option for finding solutions to problems that kept you from achieving your goals?** | | | | 🔾^1^ | | 🔾^2^ | | | | 🔾^3^ | | | | 🔾^4^ | | | | 🔾^5^ | | | | 🔾 | |
|  | | | | | | | | | | | | | | | | | | | | | | | | |
|  | | | | | | | | | | | | | | | | | | | | | | | | |
| **To what extent do you agree with the following statements?** | | | | | | | | | | | | | | | | | | | | | | | | |
|  | | | | | **Totally disagree** | | | **Disagree** | | | | **No opinion** | | | | | **Agree** | | | | | **Totally Agree** | | |
| 15 | I would recommend the app to others. | | | | 🔾^1^ | | | 🔾^2^ | | | | 🔾^3^ | | | | | 🔾^4^ | | | | | 🔾^5^ | | |
| 16 | **I plan to use the app for at least 4 more weeks.** | | | | 🔾^1^ | | | 🔾^2^ | | | | 🔾^3^ | | | | | 🔾^4^ | | | | | 🔾^5^ | | |
| 17 | **The app stimulated me to take better care of my teeth.** | | | | 🔾^1^ | | | 🔾^2^ | | | | 🔾^3^ | | | | | 🔾^4^ | | | | | 🔾^5^ | | |
| 18 | **I have truthfully indicated whether I did or did not do my tasks (brushing, proxy brushes, and mouth rinse).** | | | | 🔾^1^ | | | 🔾^2^ | | | | 🔾^3^ | | | | | 🔾^4^ | | | | | 🔾^5^ | | |
| 19 | **I have truthfully indicated which factors influenced whether or not I achieved my goals.** | | | | 🔾^1^ | | | 🔾^2^ | | | | 🔾^3^ | | | | | 🔾^4^ | | | | | 🔾^5^ | | |
| 20 | The app is well designed. | | | | 🔾^0^ | | | 🔾^2,5^ | | | | 🔾^5^ | | | | | 🔾^7,5^ | | | | | 🔾^10^ | | |
| 21 | **The apps layout looks good.** | | | | 🔾^1^ | | | 🔾^2^ | | | | 🔾^3^ | | | | | 🔾^4^ | | | | | 🔾^5^ | | |
| 22 | **The animations appeal to me.** | | | | 🔾^1^ | | | 🔾^2^ | | | | 🔾^3^ | | | | | 🔾^4^ | | | | | 🔾^5^ | | |
| 23 | **The app looks childish.** | | | | 🔾^5^ | | | 🔾^4^ | | | | 🔾^3^ | | | | | 🔾^2^ | | | | | 🔾^1^ | | |
|  | | | | | | | | | | | | | | | | | | | | | | | | |
| 24 | What score (scaling from 1 to 10) would you give the app? | | | | | | | | | | | | | | | | | | | | | | | |
| 1  🔾 | | 2  🔾 | 3 🔾 | 4  🔾 | | 5  🔾 | | | | 6  🔾 | | | 7  🔾 | | | 8  🔾 | | | | 9  🔾 | | | | 10  🔾 |
|  | | | | | | | | | | | | | | | | | | | | | | | | |
| 25 | Do you have any tips on improving the app, or any questions or remarks about this research? If so, please write them here. | | | | | | | | | | | | | | | | | | | | | | | |
| ………………………………………………………………………………………………….……………  ………………………………………………………………………………………………….……………  ………………………………………………………………………………………………….……………  ………………………………………………………………………………………………….……………  ………………………………………………………………………………………………….……………  ………………………………………………………………………………………………….……………  ………………………………………………………………………………………………….…………… | | | | | | | | | | | | | | | | | | | | | | | | |
|  | | | | | | | | | | | | | | | | | | | | | | | | |
|  | | | | | | | | | | | | | | | | | | | | | | | | |
| **To what extent do you agree with the following statements?** | | | | | | | | | | | | | | | | | | | | | | | | |
|  | | | | | **Totally disagree** | | | | **Disagree** | | | | | **No opinion** | | | | **Agree** | | | **Totally Agree** | | | |
| 26 | I found the app unnecessarily complex | | | | 🔾^10^ | | | | 🔾^7,5^ | | | | | 🔾^5^ | | | | 🔾^2,5^ | | | 🔾^0^ | | | |
| 27 | I thought the app was easy to use | | | | 🔾^0^ | | | | 🔾^2,5^ | | | | | 🔾^5^ | | | | 🔾^7,5^ | | | 🔾^10^ | | | |
| 28 | I think that I would need the support of a technical person to be able to use this app | | | | 🔾^10^ | | | | 🔾^7,5^ | | | | | 🔾^5^ | | | | 🔾^2,5^ | | | 🔾^0^ | | | |
| 29 | I found the various functions in this app were well integrated | | | | 🔾^0^ | | | | 🔾^2,5^ | | | | | 🔾^5^ | | | | 🔾^7,5^ | | | 🔾^10^ | | | |
| 30 | I thought there was too much inconsistency in this app | | | | 🔾^10^ | | | | 🔾^7,5^ | | | | | 🔾^5^ | | | | 🔾^2,5^ | | | 🔾^0^ | | | |
| 31 | I would imagine that most people would learn to use this app very quickly | | | | 🔾^0^ | | | | 🔾^2,5^ | | | | | 🔾^5^ | | | | 🔾^7,5^ | | | 🔾^10^ | | | |
| 32 | I found the app very cumbersome to use | | | | 🔾^10^ | | | | 🔾^7,5^ | | | | | 🔾^5^ | | | | 🔾^2,5^ | | | 🔾^0^ | | | |
| 33 | I felt very confident using the app | | | | 🔾^0^ | | | | 🔾^2,5^ | | | | | 🔾^5^ | | | | 🔾^7,5^ | | | 🔾^10^ | | | |
| 34 | I needed to learn a lot of things before I could get going with this app | | | | 🔾^10^ | | | | 🔾^7,5^ | | | | | 🔾^5^ | | | | 🔾^2,5^ | | | 🔾^0^ | | | |

***References:***

^1^ *IOS: International Organisation for Standardisation. Quality model, ISO/IEC 9126-1:2001.*

^2^ *Brooke, J. SUS-A quick and dirty usability scale. Usability evaluation in industry, 1996;189(194), 4-7.*

^3^ *Lewis, JR., Sauro, J. The factor structure of the system usability scale. Human centered design, 2009; 94-103.*

^4^ *Sauro, J. A practical guide to the System Usability Scale: Background, benchmarks, & best practices. Denver, CO: Measuring Usability LLC, 2011.*
