# Supplementary material for: Study protocol of a randomized controlled trial to test the effect of a smartphone application on oral-health behavior and oral hygiene in adolescents with fixed orthodontic appliances
Source: BMC Oral Health. 2018 Feb 7;18:19. doi: 10.1186/s12903-018-0475-9 (PMC5803887; doi:10.1186/s12903-018-0475-9)
Supplement: Supplementary file 5 — Figure SPIRIT Schedule of enrolment, interventions, and assessments. (DOCX 16 kb) [file 12903_2018_475_MOESM5_ESM.docx]

Additional file 5:

Figure 3. SPIRIT Schedule of enrolment, interventions, and assessments

|  | **STUDY PERIOD** | | | |
| --- | --- | --- | --- | --- |
|  | **Enrolment** | **Allocation** | **Post-allocation** | |
| **TIMEPOINT**** | ***-T_1_*** | **T*_0_*** | ***T_1_*** | ***T_2_*** |
| **ENROLMENT:** |  |  |  |  |
| **Eligibility screen** | X |  |  |  |
| **Informed consent** | X |  |  |  |
| **Randomization by independent researcher** | X |  |  |  |
| **Allocation (after baseline assessments)** |  | X |  |  |
| **INTERVENTIONS:** |  |  |  |  |
| ***[Intervention ‘White Teeth’ app]*** |  |  |  |  |
| **ASSESSMENTS:** |  |  |  |  |
| ***[Demographic and background variables]*** | X | X |  |  |
| ***[Primary outcome variables: Oral health behavior, Plaque and bleeding score.]*** |  | X | X | X |
| ***[Secondary outcomes: psychosocial factors of oral health behavior]*** |  | X | X | X |
| ***[Process evaluation]*** |  |  |  | X |
