# Supplementary material for: Study protocol of a randomized controlled trial to test the effect of a smartphone application on oral-health behavior and oral hygiene in adolescents with fixed orthodontic appliances
Source: BMC Oral Health. 2018 Feb 7;18:19. doi: 10.1186/s12903-018-0475-9 (PMC5803887; doi:10.1186/s12903-018-0475-9)
Supplement: Supplementary file 7 — A copy of the proof of funding. (PDF 292 kb) [file 12903_2018_475_MOESM7_ESM.pdf]

De Boelelaan 1117
1081 HV Amsterdam

postbus 7057
1007 MB Amsterdam

telefoon 020 444 4444

www.VUmc.nl

Medisch Ethische Toetsingscommissie
VU medisch centrum
voorzitter: prof. dr. J.A. Rauwerda
intern postadres: BS7, kamer H-565
telefoon: 020 - 44 45585
e-mail: metc@vumc.nl
website: www.vumc.nl/metc

prof. dr. C. van Loveren
ACTA, afd Cariologie Endodontologie Pedodontologie
Gustav Mahlerlaan 3004
1081 LA AMSTERDAM

VUmc

onderwerp	ons kenmerk	datum
niet-WMO advies	2016.162	18 mei 2016

Geachte heer van Loveren,

Het Dagelijks Bestuur van de Medisch Ethische Toetsingscommissie VU medisch centrum heeft uw onderzoek **De WITGEBIT App ter bevordering van de mondhygiëne** besproken in de vergadering van 12/04/2016.

Het onderzoek valt niet onder de reikwijdte van de Wet Medisch-wetenschappelijk Onderzoek met mensen (WMO).

Het oordeel is gebaseerd op de volgende documenten:

A1	aanbiedingsbrief	d.d. 4-4-2016
A1	commentaar METc	d.d. 20-4-2016
A1	reactie op commentaar METc	d.d. 17-5-2016
B25	privacyverklaring	getekend d.d. 4-4-2016
C1	onderzoeksprotocol	versie 1 d.d. 4-4-2016
E11	informatiebrief	versie 1 d.d. 4-4-2016 ouder/verzorger
E11	informatiebrief	versie 1 d.d. 4-4-2016 deelnemer
E2	toestemmingsverklaring	versie 1 d.d. 4-4-2016 deelnemer
E2	toestemmingsverklaring	versie 1 d.d. 4-4-2016 ouder/verzorger
F1	vragenlijst	versie 1 d.d. 4-4-2016 deel 1
F1	vragenlijst	versie 1 d.d. 4-4-2016 deel 2
F1	vragenlijst	versie 1 d.d. 4-4-2016 deel 3

Het Dagelijks Bestuur van de Medisch Ethische Toetsingscommissie VU medisch centrum wijst u erop dat hoewel het ingediende onderzoek niet onder de reikwijdte van de WMO valt, andere wet- en regelgeving (mogelijk) wel van toepassing is, waaronder:

- WGBO (Wet Geneeskundige BehandelingsOvereenkomst);
- WBP (Wet Bescherming Persoonsgegevens), zie www.cbpweb.nl;
- Code Goed Gedrag (Gedragscode gezondheidsonderzoek: gebruik medische gegevens in wetenschappelijk onderzoek), zie www.federa.org;
- Code Goed Gebruik (Gedragscode Verantwoord omgaan met lichaamsmateriaal ten behoeve van wetenschappelijk onderzoek, 2011), zie www.federa.org;
- Reglement VUmc Nader gebruik lichaamsmateriaal, zie KwaliteitsNet VUmc, document 046913, versie 2;

- Overdrachtsovereenkomst van lichaamsmateriaal, zie http://www.vumc.nl/afdelingen/METc/wetgeving/lichaamsmateriaal/;
- Biobanken: Tijdelijke regeling 'de novo' biobanken (METc VUmc 2012), zie http://www.vumc.nl/afdelingen/METc/wmo-oordeel/soorten_onderzoek/tijdelijke_regeling_biobanken/;
- WBO (Wet Bevolkings Onderzoek), zie http://www.vumc.nl/afdelingen/METc/wetgeving/wetbevolkingsonderzoek/.

To whom it may concern

We are pleased to confirm that the Medical Research Involving Human Subjects Act (WMO) does not apply to the above mentioned study and that an official approval of this study by our committee is not required.

The Medical Ethics Review Committee of VU University Medical Center is registered with the US Office for Human Research Protections (OHRP) as IRB00002991. The FWA number assigned to VU University Medical Center is FWA00017598.

Met vriendelijke groet,
namens de Medisch Ethische Toetsingscommissie VU medisch centrum,

0.0.

prof. dr. J.A. Rauwerda, voorzitter

c.c.: J.F.M. Scheerman / j.f.m.scheerman@acta.nl
