# Supplementary material for: Study protocol of a randomized controlled trial to test the effect of a smartphone application on oral-health behavior and oral hygiene in adolescents with fixed orthodontic appliances
Source: BMC Oral Health. 2018 Feb 7;18:19. doi: 10.1186/s12903-018-0475-9 (PMC5803887; doi:10.1186/s12903-018-0475-9)

ACTA

ACADEMIC CENTRE FOR DENTISTRY AMSTERDAM

Division	: Research Institute ACTA
Phone	: +31 20 5980641
Contact	: Dr. TJM. van Steenbergen
Email	: t.van.steenbergen@acta.nl
Date	:January 10, 2018
Reference	: MvS
Subject	: Trial to Test the Effect of a Smartphone Application on Oral-health behavior and Oral Hygiene in Adolescents

Liam Messin PhD
Editor
BMC-series *Journals*
BioMed Central
Floor 6, 236 Gray's Inn Road
London, WC1X 8HL
UK

Dear dr. Messin,

Herewith I declare that the trial related to the study protocol "Study Protocol of a Randomized Controlled Trial to Test the Effect of a Smartphone Application on Oral-health behavior and Oral Hygiene in Adolescents with Fixed Orthodontic Appliances" (OHEA-D-17-00008) will be financed by the Academic Centre for Dentistry Amsterdam (ACTA), University of Amsterdam and Vrije Universiteit Amsterdam, and InHolland University of applied sciences.

Your sincerely,

Dr. T.J.M. van Steenbergen
Research Coordinator ACTA

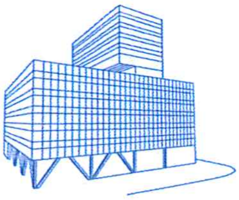

Supplement: Supplementary file 8 — A copy of the proof ethics (METC protocol. nr 2016.162). PDF (42 kb) [file 12903_2018_475_MOESM8_ESM.pdf]
